# Sample Entropy Computation on Signals with Missing Values

**DOI:** 10.3390/e26080704

**Published:** 2024-08-19

**Authors:** George Manis, Dimitrios Platakis, Roberto Sassi

**Affiliations:** 1 Department of Computer Science and Engineering, University of Ioannina, 45500 Ioannina, Greece; d.platakis@uoi.gr; 2 Dipartimento di Informatica, Università degli Studi di Milano, 20133 Milano, Italy

**Keywords:** sample entropy, missing values, vector based selection algorithm, interpolation, deletion

## Abstract

Sample entropy embeds time series into m-dimensional spaces and estimates entropy based on the distances between points in these spaces. However, when samples can be considered as missing or invalid, defining distance in the embedding space becomes problematic. Preprocessing techniques, such as deletion or interpolation, can be employed as a solution, producing time series without missing or invalid values. While deletion ignores missing values, interpolation replaces them using approximations based on neighboring points. This paper proposes a novel approach for the computation of sample entropy when values are considered as missing or invalid. The proposed algorithm accommodates points in the m-dimensional space and handles them there. A theoretical and experimental comparison of the proposed algorithm with deletion and interpolation demonstrates several advantages over these other two approaches. Notably, the deviation of the expected sample entropy value for the proposed methodology consistently proves to be lowest one.

## 1. Introduction

An artifact refers to any unintended or undesirable distortion or alteration introduced into the signal during its acquisition, processing, transmission, or recording. Artifacts can occur for various reasons such as equipment limitations, interference, noise, or errors in measurement or data processing.

In this paper, we are not interested in how those artifacts have been introduced into the signal or how to identify them. We consider artifacts as already marked sample points, which have been detected by an automatic or manual procedure. We will handle them as *missing values*. Even ideally detected and marked, missing values are still an issue when extracting sensitive information from physiological systems and special care has to be taken in order to limit their effect on the computed signal characteristics. An example of a database in which artifacts have been marked as missing values and have been replaced with zeros is [1].

Sample entropy [2,3] is a measure employed in signal processing and time series analysis to quantify the complexity or irregularity of a time series. It provides a way to characterize the predictability or regularity of a signal. A higher sample entropy value indicates greater complexity or irregularity in the time series, suggesting that the data are less predictable and the system more complex. Sample entropy is widely used in various fields such as biomedical signal processing, including Electroencephalogram (EEG) [4,5] and Heart Rate Variability (HRV) [6,7] analysis, generally studying the dynamics of physiological phenomena. It is also used in other scientific fields like economics [8] or physics [9]. The main drawback of sample entropy is the high computational complexity, which can be very crucial when the examined time series is long. Fast algorithms reducing the computation time of sample entropy have been proposed and include [10,11].

Sample entropy is an entropy definition that embeds the time series into a high-dimensional space. Vectors of size *m* are produced from the original data points. Thus, samples of the time series are examined in the context of a neighborhood rather than individually, retaining the valuable inter-sample relationship, i.e., how the signal changes or varies between consecutive or neighboring samples. Contrary to Shannon entropy, which is calculated based on a probability distribution and examines samples individually, sample entropy takes into account the order of the samples. For example, sample entropy reports different values before and after a time series is shuffled or sorted. How Shannon entropy can organize the samples is an interesting subject, but also another topic of discussion. What we need to emphasize here is the importance of the order of the samples in sample entropy.

Two different approaches for handling missing values can be considered: (a) remove missing values before embedding the time series and (b) generate artificial values to replace them. Removing the missing values destroys the inter-sample relationship, a fundamental concept and the main reason to embed signals in a higher dimensional space. On the other hand, many imputation techniques have been proposed such as interpolation [12,13,14], KNNI [15], SVDI [16], Global Most Common attribute (GMC) [17], Regularized Expectation-Maximization (EM) [18], SVM Regression [19], Local Least Squares Imputation (LLSI) [20], and Bayesian PCA (BPCA) [21]. In this paper, the imputation technique we will focus on is the interpolation. Each approach has a critical drawback, something that acted as a motivation for our research and the proposed algorithm.

To address the problem, a new strategy is investigated. Rather than preprocessing the signal by removing or replacing the missing values, the new algorithm accommodates vectors with missing values in the *m*-dimensional space and handles them there. Vectors with missing values are excluded from the rest of the computation in a vector-based, rather than in a single-dimension point-based, decision. We should note that all vectors, on which the estimation is based, are parts (of size *m*) of the original time series. The proposed algorithm also finds application in approximate entropy [22], a definition of entropy on which sample entropy was based, as well as on every definition of entropy, which embeds signals into higher dimensional spaces, e.g., permutation entropy [23], multiscale entropy [24] and bubble entropy [25].

The rest of the paper is structured as follows. Section 2 briefly outlines sample entropy. The proposed algorithm and a theoretical comparison with other approaches is the subject of Section 3. In Section 4, the experimental results express the superiority of the suggested approach, by calculating the missing values due to the diversion between the computed and expected value of the sample entropy, employing a publicly available data set with 54 Holter recordings of subjects in normal sinus rhythm [26]. The last section concludes this work.

## 2. Sample Entropy

In this section, we provide a detailed description of the definition of sample entropy [2,3]. Sample entropy is calculated based on the probability that patterns of data points within a specified length and tolerance level will repeat in a time series. It measures the likelihood that similar patterns will remain similar when the length of the patterns is increased by one data point.

Sample entropy requires the estimation of two parameters: (a) the size of the embedding space (*m*), i.e., how many samples will be included in a vector, or in other words, what the dimension of the space in which the time series will be embedded, and (b) the threshold distance (*r*), a distance under which two points are considered as *similar*. Please note that the value of *r* is multiplied by the standard deviation of the signal before being used, r:=r∗std(x), for scaling and normalization.

Two points $p_{i}^{\left(m\right)},p_{j}^{\left(m\right)}$ in the *m*-dimensional space (i.e., vectors) are defined as similar when:

(1) $$∥p_{i}^{\left(m\right)}-p_{j}^{\left(m\right)}{∥}_{\infty }=\max_{1\leq k\leq m}\left|p_{i,k}-p_{j,k}\right|<r$$

The time series $x\;=\;x_{1},x_{2},\ldots ,x_{N}$ is embedded into the *m*-dimensional space, creating vectors from consecutive points. For each data point $x_{i}$, a vector $X_{i}^{\left(m\right)}$ is constructed, formed by *m* consecutive data points starting from $x_{i}$, i.e., the *i*th vector is: $X_{i}^{\left(m\right)}\;=\;\left(x_{i},x_{i+1},\cdots x_{i+m-1}\right)$. This process is repeated for each data point in the time series, resulting in a series of vectors:

(2) $$X^{\left(m\right)}\;=\;X_{1}^{\left(m\right)},X_{2}^{\left(m\right)},\cdots X_{N-m+1}^{\left(m\right)}.$$

The choice of both *m* and *r* is crucial, as it affects the sensitivity of the analysis. Typical values for *m* and *r* are m=2,r=0.2, while the values of m=1 and m = 3 are also used. Computimg sample entropy for m=4 is not common.

Each vector $X_{i}^{\left(m\right)}$ is compared with every other vector $X_{j}^{\left(m\right)}$, where i>j is used to assess their similarity. Usually, the probability of two vectors $X_{i}^{\left(m+1\right)}\;=\;\left(x_{i},x_{i+1},\cdots x_{i+m}\right)$ and $X_{j}^{\left(m+1\right)}\;=\;\left(x_{j},x_{i+j},\cdots x_{j+m}\right)$ being similar is denoted as $B_{m+1}$, while the probability the corresponding vectors $X_{i}^{\left(m\right)}\;=\;\left(x_{i},x_{i+1},\cdots x_{i+m-1}\right)$ and $X_{j}^{\left(m\right)}\;=\;\left(x_{j},x_{i+j},\cdots x_{j+m-1}\right)$ are similar is denoted as $A_{m}$.

For a time series *x*, sample entropy is the negative natural logarithm of the ratio of $A_{m}$ over $B_{m+1}$:

(3) $$SampEn\left(x,m,r\right)\;=\;-\ln\left(\frac{A_{m}}{B_{m+1}}\right).$$

When $B_{m+1}\;=\;0$, sample entropy is defined as follows:

(4) $$SampEn\left(x,m,r\right)\;=\;-\ln\left(A_{m}\right).$$

## 3. Deletion, Interpolation, and the Proposed Algorithm

Missing values can be ignored, replaced, or handled as missing. The proposed algorithm selects the last alternative.

### 3.1. Deletion

A straightforward solution for the missing values problem is simply to delete them. It offers a quick, easy, and fair solution for entropy definitions like Shannon entropy, which estimates entropy in a single dimension. However, when moving to an *m*-dimensional space, its sole advantages lie in ease and speed.

**Figure 1:**
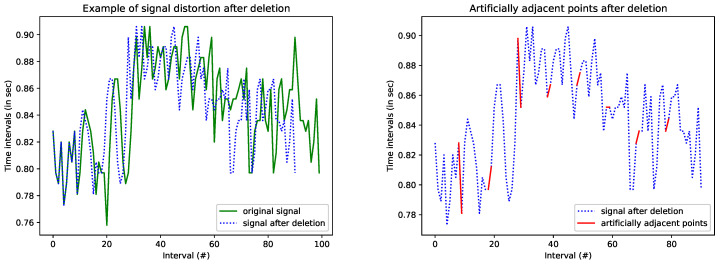
An example of deletion. The subfigure on the **left** shows the signal distortion after considering and removing 8 out of the 100 values as missing. Subfigure on the **right** shows points which were not adjacent in the input signal, but appear as adjacent after deletion.

In Figure 1, on the left hand side of the figure, part of an HRV series obtained from an Holter recording from a healthy subject (origin of the Holter recording [26]) is shown in green. Eight samples out of the one hundred samples of the original series were randomly selected to be considered as missing. We removed those eight samples and shifted the remaining ones left to remove gaps. The new series, produced after the deletion, was eight samples smaller than the initial series. The blue dotted line shown on the figure is the time series after having removed those samples and after having shifting the remaining samples left. The distortion of the signal is obvious.

In the same figure, on the right hand side, the regions in red indicate points of the signal which, after deletion became adjacent, while they were not adjacent in the original signal. Sometimes, the distance between them is small (small red segments), indicating a possibly small distortion, and sometimes it is large (larger red segments), implying a possibly more significant distortion. In any case, distortion is inevitable. Computation of entropy based on such segments is based on artifacts and generates inaccurate or even erroneous estimations.

### 3.2. Interpolation

A second solution to the the problem of missing values is to predict them and replace them with the predicted values, employing popular interpolation methods.

**Figure 2:**
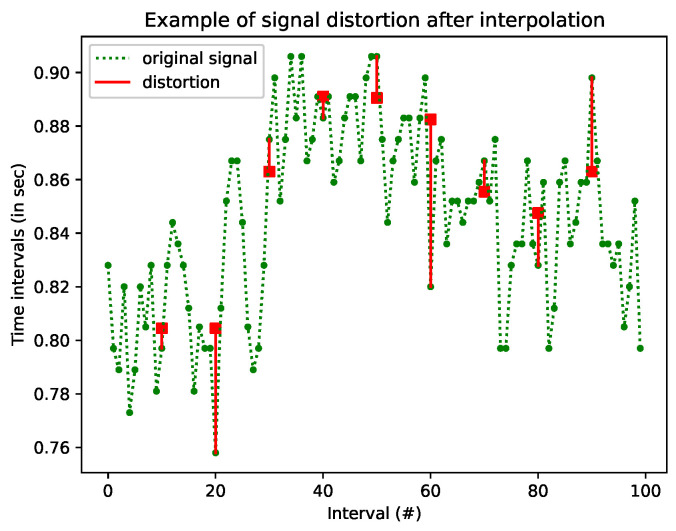
An example of interpolation. The green line is part of HRV series obtained from a Holter recording. Red circles are points computed with linear interpolation based on neighboring points. The red lines show the diversion between the real value and the interpolated one.

In Figure 2, the same part of the signal is depicted. Similar to the procedure we followed in the case of deletion, a number of samples were randomly selected to be considered as missing. Those samples were not removed from the series, as happened in the deletion, but were replaced with values computed after (linear) interpolation. The original samples are marked with green bullets. The interpolated values are marked with red squares. The distance between the original values and the interpolated ones is shown with a red line. The length of the red lines indicate the distortion of the series A number of missing values were artificially inserted again, before (linear) interpolation was used to replace them. Red squares indicates the interpolated values, while the original samples of the signal are marked with green bullets. The diversion is shown with a red line.

One can easily note that the predicted value is not always close to the original one, causing significant information distortion in some circumstances. Distortion that affects the estimated entropy of the signal, by sometimes increasing it and sometimes reducing it, is based on a flawed assumption in any case. Most of the time, the interpolated values reduce the variability, making the signal more predictable, as interpolation is a result of prediction. This confirms the theoretical expectation that interpolation decreases variability and increases predictability, two properties highly connected to entropy.

We can reach to the conclusion that, even though interpolation offers an obvious solution to the missing values problem, the fundamental concept of interpolation works against the informativeness of the signal, against the effectiveness of the estimator, and motivates us to search for an alternative, even better, solution customized on sample entropy.

### 3.3. Vector-Based Selection: The Proposed Algorithm

Rather than excluding missing points from the initial single-dimensional signal, the proposed methodology embeds the signal into the *m*-dimensional space first and then excludes the *m*-dimensional points (vectors) with missing values from the *m*-dimensional time series.

Suppose we need to determine sample entropy on the time series x = x${\;\;}_{1}$,x${\;\;}_{2}$,⋯,x${\;\;}_{N}$ with parameters *m* and *r*. The series x is embedded into the *m*-dimensional space, producing the time series V = v${\;\;}_{1}$,v${\;\;}_{2}$,⋯,v${\;\;}_{N-m}$. Vectors with missing values are not included in V. We will use the notation V${\;\;}^{B_{m+1}}$ to symbolize a list with vectors in an embedding space of size m+1 and V${\;\;}^{A_{m}}$ to symbolize a list with vectors in an embedding space of size *m*, according to the notation used in Section 2. We keep the notation v${\;\;}^{\left(m\right)}$ and v${\;\;}^{\left(m+1\right)}$ to symbolize vectors of sizes *m* and m+1, respectively. Algorithm 1 computes V${\;\;}^{B_{m+1}}$ and V${\;\;}^{A_{m}}$:
**Algorithm 1** Vector selection for embedding spaces with dimensions *m* and m+1.# *V*${\;\;}^{B_{m+1}}$*: list of vectors of size* *m* *+ 1*
V${\;\;}^{B_{m+1}}$ = **[]**# *V*${\;\;}^{A_{m}}$*: list of vectors of size m*V${\;\;}^{A_{m}}$ = **[]**# *for every vector with size m + 1***for** i **in** [0…N−m]:          v${\;\;}_{i}^{\left(m+1\right)}$ = (x${\;\;}_{i}$, x${\;\;}_{i+1}$, …, x${\;\;}_{i+m}$)          v${\;\;}_{i}^{\left(m\right)}$ = (x${\;\;}_{i}$, x${\;\;}_{i+1}$, …, x${\;\;}_{i+m-1}$)          # *check for missing values in it*          **if not** "missing" **in** v${\;\;}_{i}^{\left(m+1\right)}$:                    # *if no missing values in it*                    # *consider it a valid vector*                    V${\;\;}^{B_{m+1}}$**.add**(v${\;\;}_{i}^{\left(m+1\right)}$)                    V${\;\;}^{A_{m}}$**.add**(v${\;\;}_{i}^{\left(m\right)}$)

Obviously, vectors v${\;\;}_{i}^{\left(m\right)}$ do not contain missing values, as the vectors v${\;\;}_{i}^{\left(m+1\right)}$ do not contain missing values.

Next, each vector v${\;\;}_{i}^{\left(m+1\right)}$ is compared with every other vector v${\;\;}_{j}^{\left(m+1\right)},i>j$ to assess their similarity. The probability two vectors of size m+1 to be similar is denoted as B${\;\;}_{\left(m+1\right)}$. The probability the corresponding vectors in V${\;\;}^{\left(m\right)}$ to be similar is denoted as $A_{m}$.

For the time series x, sample entropy is computed by the negative natural logarithm of the ratio of $A_{m}$ over $B_{m+1}$. We use the notation $SampEn^{*}\left(\right)$ to symbolize sample entropy computed by the proposed methodology:

        SampEn* (x,m,r) = −ln (A_*m*_/B_*m* + 1_)

### 3.4. A Theoretical Comparison of the Three Methods

Comparing the three examined approaches, i.e., interpolation, deletion, and vector-based selection, one can note that the main drawback of the latter is the possibly small number of participating vectors, when the signal is very noisy. Even though noisy signals are not a good source of information or conclusions, this is a limitation of the proposed algorithm that should be noticed.

However, when the number of vectors is sufficiently large, the proposed algorithm is theoretically expected to present better estimations than both of the other approaches. The algorithm is solely based on information from the original signal, while the other two introduce artifacts and base their estimation on distorted or synthesized information. The theoretical expectations are confirmed by our experimental results presented in the following section.

## 4. Experimental Results

In this section, we experimentally compare deletion and two interpolated methods with the proposed algorithm. The purpose of the comparison is to quantify how much each method influences the computed value of sample entropy.

We used a data set with 54 HRV series obtained from Holter recordings, approximately 24 h long (75,000 to 150,000 samples), in a normal sinus rhythm. The data set (“Normal Sinus Rhythm RR Interval Database”) is publicly available on the internet [26]. We repeated the same experiment 100 times and calculated the average results. From each recording and each repetition, we randomly selected a 2000 samples segment of the signal, which is approximately more than half an hour of recording. For each of these segments, we computed sample entropy. Then, we selected points and considered them as missing. The percentage of the missing points ranged from 1% of the length of the signal to the 10%, i.e., 20 to 200 samples. For the distorted signal, we computed the value of sample entropy for all three examined alternatives: deletion, interpolation, and the vector-based selection. Both linear and quadratic interpolations were employed, making the total number of examined cases four.

Linear interpolation was based on the following approximation: $x^{\prime }\;=\;\frac{\sum_{i=1}^{N}x_{i}}{N}$.

Quadratic interpolation was based on $x^{\prime }\;=\;\sqrt{\frac{\sum_{i=1}^{N}x_{i}^{2}}{N}}$.

A higher-order polynomial interpolation, such as cubic, or another interpolation method [12] would be unnecessarily complex for the specific problem.

In our first experiment, the selection of points was random. The rate of the missing values ranged from 1% to 10%. We selected the following pairs of sample entropy parameters: (a) m=1,r=0.2, (b) m=3,r=0.2, (c) m=2 ,r=0.2 (the typical ones), (d) m=2,r=0.15, and (e) m=2,r=0.25.

**Figure 3:**
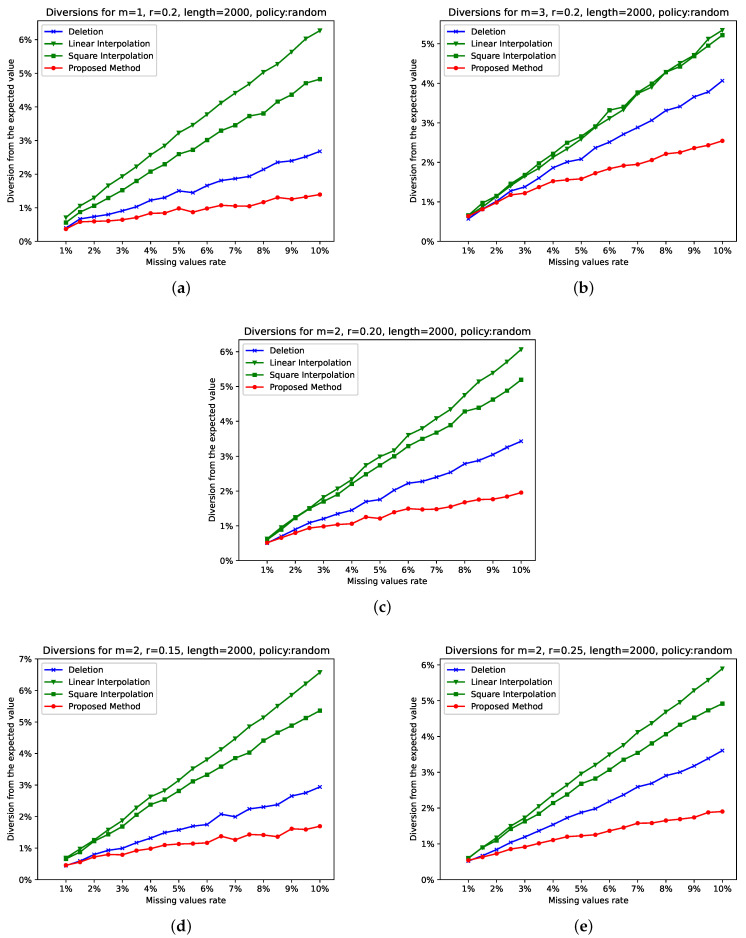
Diversions for selected values of *m* and *r*, when the selected distortion policy is “random”. Subfigure (**c**) shows the diversions for the typical values m=2,r=0.2. Subfigures (**a**,**b**), in combination with Subfigure (**c**), show how diversions are modified for all reasonable values of *m*: m=1…3. Subfigures (**d**,**e**), in combination with Subfigure (**c**) show how diversions are modified for selected values of *r*: r∈{0.15,0.20,0.25}.

We computed sample entropy before and after the distortion. The diversion from the expected value is plotted in Figure 3, as the average of 100 repetitions. In all five subfigures, the diversion computed for the proposed algorithm is not only the smallest one, but it is also significantly smaller.

We have already seen modifications on sample entropy that compute fast [11] approximations of sample entropy with the mean and root mean squared error below $10^{-3}$. In our experiments, as an evaluation metric, we used a diversion from the original method, expressed in percentages. The diversion of the proposed algorithm, for m=1 or m=2, stayed below 2% from the expected value for all missing value rates, whereas all other methods presented a considerably larger diversion. The case in which the diversion surpassed 2% is for m=3, where the error still remained much lower than that of the other two examined methodologies.

Another interesting conclusion is that deletion worked better than interpolation, verifying that the added predictability influenced the complexity of the system and the estimated entropy. Quadratic interpolation worked better than the linear one in almost all cases.

In an attempt to study a more realistic missing values distribution for HRV series, we employed the results of [27]. There, the authors built a probability mass function of the length of gaps due to missing samples from high-quality HRV series. The distribution can be well approximated with a *zipf* law, when ρ=1.5. Zipf law is given by the formula:

(5) $$zipf\left(x;\rho \right)\;=\;\frac{{\left(x-1\right)}^{-(\rho +1)}}{\zeta (\rho +1)},$$

(6) $$\mathrm{where}:\;\;\zeta \left(s\right)\;=\;\sum_{n=1}^{\infty }\frac{1}{n^{s}}.$$

**Figure 4:**
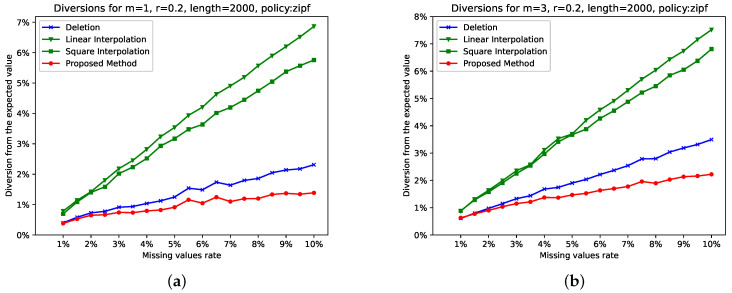

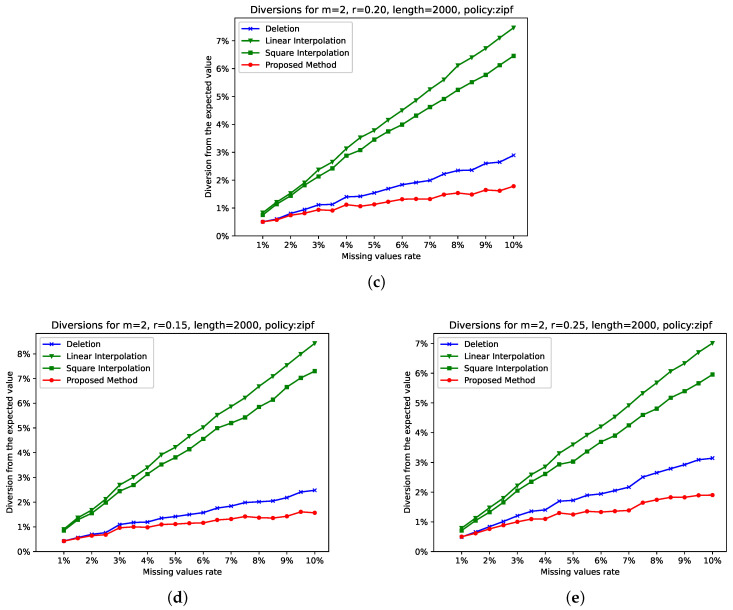
Diversions for selected values of *m* and *r*, when the selected distortion policy is “zipf”. Subfigure (**c**) shows the diversions for the typical values m=2,r=0.2. Subfigures (**a**,**b**), in combination with Subfigure (**c**), show how diversions are modified for all reasonable values of *m*: m=1…3. Subfigures (**d**,**e**), in combination with Subfigure (**c**), show how diversions are modified for selected values of *r*: r∈{0.15,0.20,0.25}.

We performed exactly the same experiments and produced the diversions depicted in Figure 4. The conclusions are similar to those extracted from Figure 3, as, again, the proposed algorithm reported the smallest diversions in all cases.

We close this section with some additional remarks. The larger the values of *m* and *r*, the larger the distance between the diversions of the proposed algorithm and the other examined methods. According to the diversion reported by the two experiments, the zipf distribution presented smaller diversions than the random one for both the proposed algorithm and deletion, while it was larger for the two interpolation methods. This observation verifies the theoretical expectation that interpolation makes the signal more predictable, reduces informativeness, and influences the estimation of entropy.

We would also like to report our experiences when we used a first order auto-regressive model to examine how parameter *a* influences the deviation from the expected value. The model we used was the following:

(7) $$x_{t}\;=\;ax_{t-1}+\epsilon_{t},$$

where $\epsilon_{t}$ is the white noise error term at time *t*. Synthetic signals were generated and missing values were added artificially, following the same procedure as we did with the Holter recordings. Values of *a* ranged from −1 to 1 and for each value of *a*, synthetic signals with 1–10% missing values were examined. Two conclusion can be reported: (a) the larger the number of missing values, the larger the deviation, as computation was based on a smaller number of vectors and (b) the minimum deviation was for a=0, i.e., white noise, as the information encrypted in the signal in this case was the minimum.

## 5. Conclusions

In this paper, we presented a new algorithm for the estimation of sample entropy, for the case in which some of the samples of the time series are considered as missing. Contrary to widely used methods, the proposed algorithm does not preprocess the input time series in order to produce a time series without missing values. The proposed algorithm embeds the time series into the *m*-dimensional space and excludes all vectors containing missing values. In this way, every non-excluded vector is formed by valid samples and can be located as a segment of *m* samples in the input time series. Ensuring that the computation of sample entropy depends only on non-distorted information is promising and was proven to be significant. We compared the proposed method with deletion and interpolation. Deletion deletes samples marked as missing, while interpolation replaces them with estimations. Both theoretical and experimental comparisons showed that the proposed algorithm outperforms the other two examined alternatives, especially the widely used and quite popular interpolation.

## Data Availability

This research has been based on a publicly available data set.
